# Comparison of real-time PCR and microscopy for malaria parasite detection in Malawian pregnant women

**DOI:** 10.1186/1475-2875-9-269

**Published:** 2010-10-06

**Authors:** Anne-Maria Rantala, Steve M Taylor, Paul A Trottman, Mari Luntamo, Bernard Mbewe, Kenneth Maleta, Teija Kulmala, Per Ashorn, Steven R Meshnick

**Affiliations:** 1 Department of International Health, University of Tampere Medical School, Tampere, Finland; 2 Department of Epidemiology, Gillings School of Global Public Health, University of North Carolina, Chapel Hill, USA; 3 Division of Infectious Diseases and International Health, Duke University Medical Center, Durham, NC, USA; 4 Division of Community Medicine, University of Malawi College of Medicine, Blantyre, Malawi

## Abstract

**Background:**

New diagnostic tools for malaria are required owing to the changing epidemiology of malaria, particularly among pregnant women in sub-Saharan Africa. Real-time PCR assays targeting *Plasmodium falciparum *lactate dehydrogenase (*pfldh*) gene may facilitate the identification of a high proportion of pregnant women with a *P. falciparum *parasitaemia below the threshold of microscopy. These molecular methods will enable further studies on the effects of these submicroscopic infections on maternal health and birth outcomes.

**Methods:**

The *pfldh *real-time PCR assay and conventional microscopy were compared for the detection of *P. falciparum *from dried blood spots and blood smears collected from the peripheral blood of 475 Malawian women at delivery. A cycle threshold (Ct) of the real-time PCR was determined optimizing the sensitivity and specificity of the *pfldh *PCR assay compared to microscopy. A real-time PCR species-specific assay was applied to identify the contribution to malaria infections of three *Plasmodium *species (*P. falciparum P. ovale *and *P. malariae*) in 44 discordant smear and *pfldh *PCR assay results.

**Results:**

Of the 475 women, *P. falciparum *was detected in 11 (2.3%) by microscopy and in 51 (10.7%) by real-time PCR; compared to microscopy, the sensitivity of real-time PCR was 90.9% and the specificity 91.2%. If a Ct value of 38 was used as a cut-off, specificity improved to 94.6% with no change in sensitivity. The real-time PCR species-specific assay detected *P. falciparum *alone in all but four samples: two samples were mixed infections with *P. falciparum *and *P. malariae*, one was a pure *P. malariae *infection and one was a *pfldh *PCR assay-positive/species-specific assay-negative sample. Of three *P. malariae *infections detected by microscopy, only one was confirmed by the species-specific assay.

**Conclusions:**

Although microscopy remains the most appropriate method for clinical malaria diagnosis in field settings, molecular diagnostics such as real-time PCR offer a more reliable means to detect malaria parasites, particularly at low levels. Determination of the possible contribution of these submicroscopic infections to poor birth outcomes and maternal health is critical. For future studies to investigate these effects, this *pfldh *real-time PCR assay offers a reliable detection method.

## Background

Malaria in pregnancy produces substantial maternal and infant morbidity and mortality in sub-Saharan Africa [1]. Compared with non-pregnant women, pregnant women - especially primigravidae - are at particularly high risk for *Plasmodium falciparum *infection. In mothers, malaria contributes to maternal anaemia and peripartum morbidity, while infants suffer from low birth weight resulting both from intrauterine growth retardation and pre-term delivery; up to 200,000 infant deaths every year are attributed to malaria in pregnancy [1]. Consequently, most malaria-endemic countries in sub-Saharan Africa administer intermittent preventive therapy in pregnancy (IPTp) with two to three doses of sulphadoxine-pyrimethamine (SP) to all pregnant women. IPTp substantially reduces malaria during pregnancy and improves maternal and infant health outcomes [2-6]. Unfortunately, parasite resistance to SP is prevalent in many African countries and endangers the drug's utility as IPTp [7,8].

Submicroscopic infections are common during pregnancy [9,10]. Molecular methods are, on the average, capable of finding approximately twice as many infections as microscopy [11]. However, the epidemiology and clinical significance of submicroscopic malaria infections during pregnancy have not been well studied.

In this cross-sectional study, two malaria diagnostic methods, a real-time PCR assay targeting the *P. falciparum *lactate dehydrogenase gene (*pfldh*) and conventional microscopy, were compared among women delivering at the local health facilities in or near Lungwena, Malawi, where malaria is holoendemic. The goals of this study were twofold: 1) to evaluate whether real-time PCR increases detection of *P. falciparum *infections in Malawian pregnant women compared to microscopy, and 2) to determine a real-time PCR cycle threshold (Ct) that optimizes sensitivity and specificity compared to microscopy. In addition, in this study a real-time PCR species-specific assay was applied to identify the contribution to malaria infections during pregnancy of non*-falciparum *species.

## Methods

### Study area and population

The samples were collected as part of the Lungwena Antenatal Intervention Study (LAIS), which is described in detail elsewhere [12]. Briefly, the LAIS enrolled 1320 women in the second trimester at a single rural health centre in the Mangochi district in southern Malawi between December 2003 and October 2006. This is a hot, dry and low-lying rural area and the population is mainly engaged in subsistence farming and fishing. Malaria is holoendemic in Malawi despite the common use of bed nets in local households. The rainy season is between December and March [13]. The women received antenatal care and IPTp with either two doses of SP, monthly SP, or monthly SP combined with two doses of azithromycin and were followed longitudinally until one month after delivery. The trial was performed according to Good Clinical Practice guidelines (ICH-GCP) and its protocol was approved by the College of Medicine Research and Ethics Committee, University of Malawi and the Ethical Committee of Pirkanmaa Hospital District, Finland.

### Sample collection and preparation

Maternal peripheral venous blood samples were collected as dried blood spots and blood films briefly after delivery from the women who gave birth at the local health facilities. Giemsa-stained thin and thick blood films were prepared and subsequently interpreted by an experienced microscopist in a local research laboratory. Thick smears were defined as negative if 200 high-power fields were free of all malaria species. Additionally, the maternal venous peripheral blood was prepared as dried blood spots: two 50 μl aliquots of blood from the same patient were applied to Whatman FTA filter paper (Whatman plc, Maidstone, UK), air-dried immediately and placed in individually sealed plastic bags with desiccant. The sample bags were stored in dry conditions at room temperature prior to transport to the University of Tampere, Finland.

### DNA extraction

Blood spots were cut from each filter paper using scissors and deposited into individual 1.5 mL tubes. Scissors were sterilized twice with 100% ethanol and flame after each sample. Genomic DNA (gDNA) was extracted from the blood spots using the BioRobot M48 workstation (Qiagen, Valencia, CA, USA). The DNA Mini M48 Kit (Qiagen) protocol "Isolation of DNA from dried blood" was followed with these exceptions: the sample disk was incubated with 760 μl of buffer MTL(1) in continuous mixing (130 rpm) at 70°C for 1 hour; and before starting the isolation programme the filter paper disk was removed from the sample solution. Genomic DNA was eluted into 100 μl of elution buffer for use in real-time PCR assays.

### Real-time PCR testing

All gDNA samples from maternal peripheral blood were amplified in an assay targeting the *P. falciparum *lactate dehydrogenase gene (*pfldh*) [14] (see Table 1 for primer and probe sequences). Reaction volumes were 25 μl each, consisting of 12.5 μl Universal PCR Master Mix (Applied Biosystems), 1 μl of gDNA, forward and reverse primers at 250 nM each, probe at 300 nM, and molecular-grade water. All reactions were run in duplicate on an ABI PRISM 7000 Real-Time System (Applied Biosystems, Foster City, CA, USA). The cycling conditions were: 50°C for 2 min, 95°C for 10 min, and 40 cycles of 95°C for 15 s followed by 60°C for 1 min. Each reaction plate included four serial dilutions (10, 1, 0.1, 0.01 ng/μl) of *P. falciparum *3D7 genomic DNA extracted from lab-cultured parasites [15] as positive controls and a negative control with molecular-grade water in place of DNA, all in duplicate. For each plate, threshold lines were set manually and mean Ct was calculated for each amplified duplicate. Samples were considered *P. falciparum*-positive if both amplification curves reached the threshold line. Reactions with only one amplification curve reaching the threshold line or with a Ct value between 39 and 40 were repeated in duplicate with a template volume of 2 μl. Ct values were plotted against the logarithmic concentrations of 3D7 control gDNA and correlation coefficient (R^2^) values and sample concentrations were calculated.

**Table 1:** Primer and probe sequences

Assay	Reagent		**Sequence**^**c**^
*Pfldh*	Primers^a^	*P. falciparum *LDH forward	ACG ATT TGG CTG GAG CAG AT
		
		*P. falciparum *LDH reverse	TCT CTA TTC CAT TCT TTG TCA CTC TTT C
	
	Probe^b^	TaqMan probe (*P. falciparum *LDH)	FAM-AGT AAT AGT AAC AGC TGG ATT TAC CAA GGC CCC A-TAMRA

*Species-specific*	Primers^a^	*P. falciparum *18 S rDNA forward	ATT GCT TTT GAG AGG TTT TGT TAC TTT
		
		*P. falciparum *18 S rDNA reverse	GCT GTA GTA TTC AAA CAC AAT GAA CTC AA
		
		*P. ovale *18 S rDNA forward	CCG ACT AGG TTT TGG ATG AAA GAT TTT T
		
		*P. ovale *18 S rDNA reverse	CAA CCC AAA GAC TTT GAT TTC TCA TAA
		
		*P. malariae *18 S rDNA forward	AGT TAA GGG AGT GAA GAC GAT CAG A
		
		*P. malariae *18 S rDNA reverse	CAA CCC AAA GAC TTT GAT TTC TCA TAA
		
		Human GAPDH forward	CCT CCC GCT TCG CTC TCT
		
		Human GAPDH reverse	GCT GGC GAC GCA AAA GA
	
	MGB probes^b^	*P. falciparum*	FAM-CAT AAC AGA CGG GTA GTC AT
		
		*P. ovale*	VIC-CGA AAG GAA TTT TCT TAT T
		
		*P. malariae*	FAM-ATG AGT GTT TCT TTT AGA TAG C
		
		Human GAPDH	VIC-CCT CCT GTT CGA CAG TCA GCC GC

*^a ^*Synthesized by MWG/Operon Biotech (High Point, NC) and re-suspended in molecular-grade water. LDH, lactate dehydrogenase. GAPDH, glyceraldehyde-3-phosphate dehydrogenase.

*^b ^*Synthesized by Applied Biosystems (Foster City, CA) and diluted in Tris-EDTA Buffer (FisherBioTech, Fair Lawn, NJ). LDH, lactate dehydrogenase. GAPDH, glyceraldehyde-3-phosphate dehydrogenase.

*^c ^*FAM and VIC denote fluorescent dyes. TAMRA denotes the quencher 6-carboxytetramethylrhodamine. MGB, minor groove binding probes.

To determine the specificity of the *pfldh *PCR assay, all samples with discordant microscopy and real-time PCR results were specified in a real-time PCR assay targeting species-specific sequences of the ribosomal DNA (rDNA) of *P. falciparum*, *P. ovale*, and *P. malariae*. Primer and probe sequences were adapted from an earlier study [16] (Table 1). The assay consisted of two parallel duplex reactions employing minor-groove binding (MGB) probes labeled with FAM or VIC fluorophores to detect *P. falciparum *and *P. ovale *in one reaction and *P. malariae *and a human gene (as a positive control) in the second [16].

### Statistical analyses

The results from *pfldh *PCR assay were compared to microscopy results. The prevalence of malaria infections by each method was compared by month. Using microscopy as the 'gold standard', we calculated sensitivity (number of true positives/(number of true positives + number of false negatives)) and specificity (number of true negatives/(number of true negatives + number of false positives)), as well as positive and negative predictive values for qualitative detection of *P. falciparum *infections by the *pfldh *PCR assay. In addition, the optimal Ct cut-off point for *pfldh *PCR assay was evaluated by calculating sensitivity and specificity for different Ct cut-off points and constructing a receiver operating characteristic (ROC) curve; the area under curve (AUC) was calculated from this figure. To analyse the effect of the detection of parasites at delivery on birth outcomes, pairwise comparisons between groups of women (based on *P. falciparum *detection) were made using the student's t-test and the chi-squared test for continuous and categorical variables, respectively. Comparisons were two-tailed, and a p-value of < 0.05 was considered significant. All statistical analyses were performed in MS Excel (v 12.2.5 (2008), Microsoft, Redmond, WA, USA).

## Results

### Study population

Of 1,320 women enrolled, 491 gave birth at the local health facilities (829 women delivered at home and were excluded from further analysis). Of these 491 participants, a further sixteen women were excluded because of the lack of dried blood spots (n = 6), microscopy data (n = 9) or both (n = 1), resulting in a study population of 475 women. The characteristics of these 475 participants are presented in Table 2.

**Table 2:** Characteristics of 475 women at enrollment and the time of delivery

Enrollment, second trimester:	n = 475
Age (years; mean, SD)	24 (6.3)
BMI (mean, SD)	21.9 (2.2)
Gravidity:	
Primigravidae (%)	28.6
Secundigravidae (%)	17.9
Multigravidae (%)	53.5
Bed net use by mother in enrollment (%)	60
HIV infection (%)	12.4
**Delivery:**	

Maternal haemoglobin (g/dL; mean, SD)	11.4 (1.9)*
Maternal anaemia** (%)	31.3*
Maternal microscopy result for malaria***:	
*P. falciparum *(%)	2.3
*P. vivax *(%)	0
*P. ovale *(%)	0
*P. malariae *(%)	0.6
Season of birth:	
Jan - March (%)	16.8
April - June (%)	29.9
July - Sept (%)	31.2
Oct - Dec (%)	22.1
Estimated gestational age of the child (weeks; mean, SD)	38.8 (1.8)

BMI, body mass index; *, n = 451; **, haemoglobin < 11.0 g/dL (WHO guideline 1972, 'Nutritional Anaemias'); ***, examination from Giemsa stained thin and thick blood film from maternal peripheral blood

### Sensitivity and specificity

**Figure 1:**
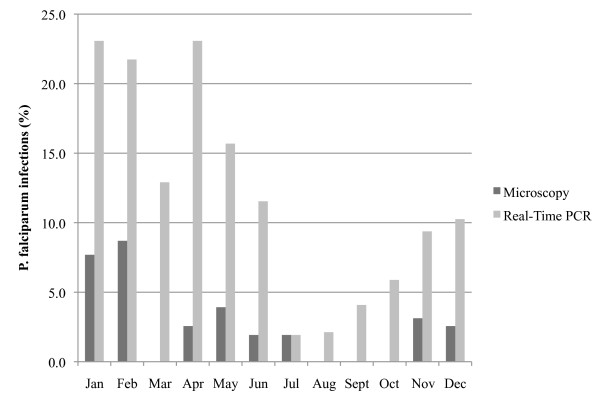
**Comparison of *P. falciparum *infections detected by microscopy and the *pfldh *real-time PCR assay at delivery by calendar months**.

Of 475 women at delivery, *P. falciparum *was detected in 11 (2.3%) by microscopy. Three additional patients had *P. malariae *monoinfections detected microscopically. In contrast, 51 (10.7%) samples were positive for *P. falciparum *by the *pfldh *PCR assay (Table 3). Of the 11 samples found to have *P. falciparum *by microscopy, 10 were positive in the *pfldh *PCR assay. Of the 464 microscopy-negative samples, 41 were positive in the *pfldh *PCR assay. Compared to microscopy, the sensitivity of real-time PCR for detection of *P. falciparum *was 90.9% and the specificity 91.2%. Submicroscopic infections were most prevalent during and after the rainy season in January-April, while microscopic malaria prevalence peaked only at the earlier part of the rainy season (January and February) (Figure 1). Thus, for example, there were 9 cases of *P. falciparum *by *pfldh *PCR, vs 1 by microscopy in April, while in July PCR and microscopy detected the same number of infections (one each).

**Table 3:** Comparison of *P. falciparum *parasites in maternal peripheral blood at delivery by the *pfldh *real-time PCR assay and microscopic thin and thick blood film examination

	Gold standard:		
**Test result:**	Microscopy		

*pfldh *real-time PCR assay	+	-	

+	10	41	51 (10.7%)

-	1	423	424 (89.3%)

	11 (2.3%)	464 (97.7%)	**Total**

			475 (100%)

### Ct cut-off points and ROC curve

**Figure 2:**
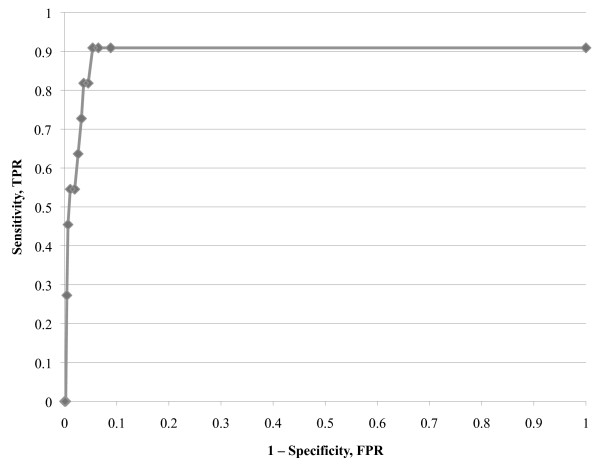
**ROC-curve calculated from the sensitivity and 1-specificity of the *pfldh *real-time PCR assay**. TPR, true positive rate, FPR, false positive rate.

The AUC of the ROC curve was 0.89. Based on the serial sensitivities and specificities at various Ct cut-off points and the ROC curve (Figure 2), a Ct cut-off of 38 optimized sensitivity and specificity at 90.9% and 94.6%, respectively. At this level, the positive predictive and negative predictive value were 28.6% and 99.8%, respectively. Notably, the ROC curve failed to reach 100 percent sensitivity because of one *pfldh *PCR-negative/microscopy-positive *P. falciparum *infection.

### Real-time PCR species-specific assay

Of the 475 women, 44 samples were analyzed in the species-specific assay, including all submicroscopic parasitaemia (41), one *pfldh *PCR-negative/microscopy-positive *P. falciparum *infection, and two *pfldh *PCR*-*negative/microscopy-positive *P. malariae *samples (Table 4). From the 41 submicroscopic samples, 40 *P. falciparum *infections were confirmed in the species-specific assay, including two samples that were mixed infections with *P. falciparum *and *P. malariae*. One sample was *pfldh *PCR-positive but negative in the species-specific assay.

**Table 4:** Detection of malaria parasites by microscopy, the *pfldh *real-time PCR assay and the species-specific real-time PCR assay

	Organism(s) detected by each method (No. of samples)		
	
**Malaria-causing species**^**a**^	Microscopy, thin and thick blood smears	Real-Time PCR, *pfldh *assay	**Real-Time PCR, species-specific assay**^**b**^
*P. falciparum*	*P. falciparum *(11)	*P. falciparum *(10)	-
		Negative (1)	Negative (1)

Non- *P. falciparum*	*P. malariae *(3)	*P. falciparum *(1)	*P. falciparum *(1)
		Negative (2)	*P. malariae *(1) Negative (1)

Negative	Negative (461)	*P. falciparum *(40)	*P. falciparum *(37) *P. falc*. + *P. malariae *(2) Negative (1)
		Negative (421)	-

Total	475	475	44

^a ^Malaria species are classified according to the diagnosis obtained from the blood smear examination.

^b ^Species-specific assay is conducted for all specimens with discordant smear and *pfldh *PCR assay results (44).

Of three patients diagnosed with *P. malariae *by microscopy, only one was positive in the species-specific assay. Furthermore, the assay detected two *P. malariae - P. falciparum *co-infections which were aparasitemic by microscopy (Table 4). Thus, although numbers were small, there is a very poor concordance between microscopy and our real-time PCR assay for the diagnosis of *P. malariae*.

### *Plasmodium falciparum *detection and birth outcomes

Of 459 newborns, 56 (12.2%) were low birth weight, defined as weight < 2500 g. There were no significant differences in the prevalence of low birth weight newborns between women with a smear-positive parasitaemia (10%), women with a submicroscopic parasitaemia (19.5%), and aparasitaemic women (11.5%; Table 5). Similarly, there were no significant differences between these groups on mean birth weight, mean gestational age at delivery, or the proportion of preterm birth.

**Table 5:** Clinical outcomes and the detection of *P. falciparum *at delivery^a^

	I	II	III	**p-value**^**b**^	
	**Microscopy-positive/*pfldh-*positive** **(n = 10)**	**Microscopy-negative/*pfldh*-positive** **(n = 41)**	**Both tests negative** **(n = 408)**	**I v. III**	**II v. III**

**Birth weight, g, mean (SD)**	2,863 (425)	2,927 (445)	2,954 (492)	0.561	0.736

**Low birth weight, % (No.)**	10 (1)	19.5 (8)	11.5 (47)	1	0.138

**Gestational age at delivery, weeks, mean (SD)**	39.2 (2.5)	38.7 (1.6)	38.8 (1.8)^c^	0.507	0.835

**Preterm delivery, % (No.)**	10 (1)	14.6 (6)	11.6 (49) ^c^	1	0.611

^a ^SD: standard deviation. Low birth weight is defined as < 2500 g, and preterm delivery as < 37 weeks.

^b ^Determined by student's t-test or chi-squared test for continuous and categorical variables, respectively.

^c ^n = 423.

## Discussion

The prevalence of submicroscopic *P. falciparum *infections was substantial among pregnant women at delivery in our cohort in Malawi. Compared with microscopy, a real-time PCR assay targeting the *pfldh *gene detected more *P. falciparum *infections: 11 (2.3%) women were microscopy-positive and 51 (10.7%) were real-time PCR positive. Compared with microscopy, the sensitivity and specificity of our real-time PCR assay were 90.9% and 91.2% respectively. Real-time PCR can be a sensitive tool to detect low-level malaria infections and thus to evaluate the effect of these infections on birth outcomes. Additionally, we found few infections with non-*falciparum *species in our pregnancy cohort.

This detection of a large number of submicroscopic infectious by real-time PCR is similar to previous studies of malaria in pregnancy [9,10]. When testing both peripheral and placental blood of pregnant women in Gabon at delivery with an assay targeting Plasmodia rDNA, [17] a real-time PCR detected three-fold more *P. falciparum *infections than microscopy (10% by microscopy and 31% by real-time PCR). Similarly, in a Kenyan study [18], four-fold more parasitaemia were detected at delivery with a real-time PCR assay compared with microscopy (37.9% and 9.4%, respectively). Submicroscopic parasitaemia detected by real-time PCR assays have been detected with similar frequency as ours at earlier gestational ages in studies in both the Sudan [19] and Mozambique [9]. These data, which indicate that the prevalence of submicroscopic infections is similar both early in pregnancy and at delivery, suggest that the good adherence to IPTp, as was common in our study, may not substantially affect the prevalence of these low-level malaria infections.

Submicroscopic infections in the general population are significant in serving as a reservoir of parasites to drive transmission intensity [11], but they usually have few consequences for the individual. In this study, parasitaemia at delivery - as detected by either blood smear or the *pfldh *PCR assay - was not associated with poor birth outcomes compared with aparasitaemic women. Previous studies have produced conflicting data on the effect of these infections on maternal or fetal health outcomes. Two earlier reports found no association between low-birth weight and submicroscopic infections in peripheral blood as detected by either rapid antigen test or PCR assays [20,21]. In contrast, real-time PCR positivity was correlated with lower birth weight in a cohort of Kenyan women, though it is unclear if this difference was accounted for by the smear-positive or by the submicroscopic infections [18]. Only one study in Gabon [17] has demonstrated a clear association between submicroscopic parasitaemia at delivery and low-birth weight, and this has yet to be confirmed in subsequent studies. Additionally, maternal anaemia has been associated with submicroscopic infections in some studies [9,17,22] but not in others [23,24]. Taken together, evidence is suggestive that submicroscopic parasitaemia at delivery is likely associated with deleterious maternal and fetal health outcomes, though the overall low prevalence of parasitaemia at delivery likely prevented the detection of deleterious consequences. More definitive characterization of these effects requires larger studies and a simple and reliable tool to detect such parasitaemia.

The *pfldh *real-time PCR assay we employed is highly sensitive and specific for *P. falciparum *infections, and offers several diagnostic advantages to conventional microscopy. Drying blood on filter paper is an effective form of DNA storage and samples prepared in this manner are cheap and safe to transport [25]. DNA extraction from dried blood spots is relatively easy and rapid to perform, and automated extraction minimizes the risk of contamination. In general, real-time PCR assays offer specific advantages over other PCR-based chemistries: they are less labor-intensive; they are performed in closed systems which minimize the risk of post-amplification contamination; assay results can be obtained in a relatively short period of time; and the assay design is automated and is well-suited to multipurpose studies [26-28]. Additionally, the *pfldh *PCR assay can be used to detect parasitaemia from peripheral blood as well as placental or cord blood.

This study has several limitations. First, though the *pfldh *PCR assay can theoretically quantify the amount of *P. falciparum *gDNA, slight variations in blood spot size and efficiency of extraction prevent reliable quantitation of parasite burden. Though it is likely that the infections detected only by the assay were, in fact, "submicroscopic", they may also be the result of poorly-sensitive microscopy. Moreover, microscopy can be an imperfect reference standard used for malaria diagnosis, and this study did not have microscopy quality control [29,30]. Additionally, the *pfldh *PCR assay, though simple and easy to perform, detects only *P. falciparum *infections. However, a species-specific assay was employed in a directed fashion to account for this, and the contribution of other species to infections at delivery was low. *Plasmodium vivax *was not addressed due to its rarity in sub-Saharan Africa [31]. Finally, due to participants who did not deliver at local health facilities, the study population may not be representative of all pregnant women with malaria at the study site, and its generalizability to other geographic areas is difficult to predict.

New diagnostic tools may be required owing to the changing epidemiology of malaria. In 1996, the prevalence of microscopy-positive *P. falciparum *malaria was 26% at delivery in a study similar to ours in Malawi [32], and in this cohort it was only 2.3%. This decrease in prevalence, which is temporally associated with increased utilization of prevention measures such as bed nets and IPTp, will impact the predictive value of blood smear results. Specifically, with our *pfldh *PCR assay as the reference, the sensitivity of microscopy was only 20% (10/51) and the positive predictive value was 91% (10/11). Further decline in prevalence will diminish the positive predictive value, necessitating a more sensitive method for detection to establish the presence of parasitaemia as a possible risk factor for poor birth outcomes.

Microscopy remains the most practical diagnostic tool in malaria-endemic areas, but its use as a 'gold standard' may produce misleading results in clinical trials [26,29,33,34]. Good-quality microscopy requires proper blood film preparation, staining, and reading by technicians with substantial expertise, and the operating characteristics of microscopy vary widely in endemic areas [2]. When compared with molecular diagnostics for malaria, microscopy readings frequently exhibit substantial discordance, producing both false-negative readings (as would be expected for submicroscopic parasitaemia) as well as false-positive readings, which can significantly impact the results of clinical trials [29]. For these reasons, molecular diagnostics may offer a more reliable means to detect malaria parasites in clinical research studies.

The added utility of more sensitive diagnostic tools such as PCR may vary seasonally according to the malaria transmission season. In this study, molecular detection of malaria yielded the greatest diagnostic value in the months at the end of the rainy season, when malaria transmission is waning, and detected few infections during the dry, low-transmission season. Because molecular testing is resource-intensive, a parsimonious approach to its application could focus on this period of declining transmission, when the added diagnostic value is highest.

Malaria control programmes that employ insecticide-treated bed nets and IPTp have significantly reduced the incidence of malaria during pregnancy, but more sensitive parasite detection methods have demonstrated a large pool of submicroscopic infections which may have negative consequences for mother and child. Determining the contribution of these submicroscopic infections to poor birth outcomes is critical. To this end, simple and reliable methods to detect these infections are essential, and we believe the *pfldh *PCR assay described herein is well-suited to future studies of submicroscopic malaria infections during pregnancy.

## Conclusions

Although microscopy remains the most appropriate method for clinical malaria diagnosis in field settings, molecular diagnostics such as real-time PCR offer a more reliable means to detect malaria parasites, particularly at low levels. Determination of the contribution of these submicroscopic infections to poor birth outcomes and maternal health is critical. For future studies to investigate these effects, the *pfldh *real-time PCR assay described herein offers a reliable detection method.

## Competing interests

The authors declare that they have no competing interests.

## Authors' contributions

AMR carried out the DNA extraction, the real-time PCR runs targeting *pfldh *gene and the statistical analyses and drafted the manuscript. SMT conducted some real time PCR reactions, contributed to the data analyses and interpretation, and wrote the paper. PAT did the real-time PCR speciation reactions. TK, ML, KM and BM ran the parent epidemiological study and contributed to data collection, data interpretation, and critical revision of the manuscript text. PA contributed to study design, data collection, data analyses and interpretation, and writing the manuscript. SRM supervised the laboratory work, contributed to the data analyses and interpretation, and wrote the manuscript. All authors read and approved the final manuscript.
